# A novel *AKT3* mutation in melanoma tumours and cell lines

**DOI:** 10.1038/sj.bjc.6604637

**Published:** 2008-09-23

**Authors:** M A Davies, K Stemke-Hale, C Tellez, T L Calderone, W Deng, V G Prieto, A J F Lazar, J E Gershenwald, G B Mills

**Affiliations:** 1 Department of Melanoma Medical Oncology, The University of Texas MD Anderson Cancer Center, 1515 Holcombe Blvd, Houston, TX 77030, USA; 2 Department of Systems Biology, The University of Texas MD Anderson Cancer Center, 1515 Holcombe Blvd, Houston, TX 77030, USA; 3 Molecular Biology and Lung Cancer Program, Lovelace Respiratory Research Institute, 2425 Ridgecrest Dr SE, Albuquerque, NM 87108, USA; 4 Department of Surgical Oncology, The University of Texas M D Anderson Cancer Center, 1515 Holcombe Blvd, Houston, TX 77030, USA; 5 Department of Pathology, The University of Texas MD Anderson Cancer Center, 1515 Holcombe Blvd, Houston, TX 77030, USA; 6 Department of Surgical Oncology and Cancer biology, The University of Texas MD Anderson Cancer Center, 1515 Holcombe Blvd, Houston, TX 77030, USA

**Keywords:** AKT, mutation, melanoma

## Abstract

Recently, a rare activating mutation of *AKT1 (E17K)* has been reported in breast, ovarian, and colorectal cancers. However, analogous activating mutations in *AKT2* or *AKT3* have not been identified in any cancer lineage. To determine the prevalence of *AKT E17K* mutations in melanoma, the most aggressive form of skin cancer, we analysed 137 human melanoma specimens and 65 human melanoma cell lines for the previously described activating mutation of *AKT1*, and for analogous mutations in *AKT2* and *AKT3.* We identified a single *AKT1 E17K* mutation. Remarkably, a previously unidentified *AKT3 E17K* mutation was detected in two melanomas (from one patient) as well as two cell lines. The AKT3 E17K mutation results in activation of AKT when expressed in human melanoma cells. This represents the first report of *AKT* mutations in melanoma, and the initial identification of an *AKT3* mutation in any human cancer lineage. We have also identified the first known human cell lines with naturally occurring *AKT E17K* mutations.

Melanoma is the most aggressive form of skin cancer, and patients with metastatic disease have a dismal prognosis that is not significantly improved with systemic therapies (Tsao *et al*, 2004). The pattern of genetic alterations in this disease suggests that activation of kinase signalling pathways has an important function in the initiation and pathophysiology of melanoma. *BRAF*, a serine-threonine kinase in the RAS/RAF/MAPK signalling cascade, is mutated in 60–70% of melanomas and, to date, represents the most common mutation in this disease (Davies *et al*, 2002; Curtin *et al*, 2005). Over 80% of the *BRAF* mutations involve a missense mutation that results in the substitution of glutamic acid for valine at amino acid 600 (V600E). Expression of this mutant form of BRAF *in vitro* results in constitutive activation of MAPK (Davies *et al*, 2002). The PI3K/AKT pathway may also have an important function in melanoma. *NRAS*, which activates both the RAS/RAF/MAPK and the PI3K/AKT signalling pathways, is mutated in approximately 15% of melanoma tumours and cell lines (Goydos *et al*, 2005; Goel *et al*, 2006). Loss of function of the tumour suppressor PTEN, which results in activation of the PI3K/AKT pathway, occurs in 10–30% of melanomas, frequently concurrent with activating *BRAF* mutations (Tsao *et al*, 1998; Zhou *et al*, 2000; Haluska *et al*, 2006). Activating mutations of *PIK3CA*, which encodes the p110*α* catalytic subunit of PI3Ks, have been detected with significant frequency in colon, breast, and ovarian cancers, but appear to be rare in melanoma (1.5%) (Curtin *et al*, 2006; Omholt *et al*, 2006).

AKT/PKB, a serine-threonine kinase, is a key downstream effector of PI3K-mediated signalling (Downward, 2004). Recently, a point mutation in *AKT1* was identified in breast, ovarian, and colon cancers (Carpten *et al*, 2007). This mutation (*E17K*) increases recruitment of AKT1 to the membrane, increases AKT1 phosphorylation and activity, transforms Rat1 fibroblasts, and induces leukaemia in transgenic mice (Carpten *et al*, 2007). No melanoma specimens were examined in that study. To determine whether *AKT E17K* mutations occur in melanoma, we used a mass spectroscopy-based approach designed to detect single-nucleotide polymorphisms (SNPs) to determine the frequency of this activating mutation in *AKT1*, *AKT2*, and *AKT3* isoforms in a large panel of human melanoma specimens and human cell lines.

## Materials and methods

### Clinical specimens

The OCT-embedded frozen clinical specimens were obtained from the Melanoma Informatics, Tissue Resource and Pathology Core at The University of Texas MD Anderson Cancer Center under an Institutional Review Board approved protocol (PI- JEG). The H&E slides were reviewed by an experienced dermatopathologist (VGP or AJFL) to identify specimens with regions enriched for viable tumour cells. These slides were used as a guide to macrodissect the OCT block and isolate the selected tumour-enriched region. Ten- to twenty-micrometre shears were prepared at −20°C and then were stored at −80°C for molecular studies. An additional H&E slide was made after all shears were prepared and was reviewed by an experienced dermatopathologist to confirm >70% tumour content in the section used. Genomic DNA was isolated using the QIAmp DNA Mini kit (Qiagen, Valencia, CA, USA) according to the manufacturer's guidelines.

### Cell lines

Human melanoma cell lines were grown under normal tissue culture conditions in RPMI supplemented with fetal calf serum. Cell pellets were isolated, and DNA was extracted using the QIAmp DNA Mini kit (Qiagen) according to the manufacturer's guidelines.

### Mass spectroscopy-based *AKT* mutation detection

A mass spectroscopy-based approach evaluating SNPs was used to detect the *AKT1 E17K* mutation, and mutations in the equivalent sites of *AKT2* and *AKT3* (Thomas *et al*, 2007). Polymerase chain reaction (PCR) and extension primers for *AKT1*, *AKT2*, and *AKT3* were designed using Sequenom Inc. (San Diego, CA, USA) Assay Design. PCR-amplified DNA was cleaned using EXO-SAP (Sequenom), primer extended by IPLEX chemistry, desalted using Clean Resin (Sequenom), and spotted onto Spectrochip matrix chips using a nanodispenser (Samsung). Chips were run in duplicate on a Sequenom MassArray MALDI-TOF MassArray system. Sequenom Typer Software and visual inspection were used to interpret mass spectra. Reactions where more than 15% of the resultant mass ran in the mutant site in both reactions were scored as positive. All mutations were confirmed by Sanger sequencing in the MD Anderson Cancer Center Support Grant-supported sequencing core.

### Plasmids

Human *AKT3* cDNA cloned into the pcDNA3 vector with a HA-tag at the N-terminus was generously provided by Dr Yiling Lu (MD Anderson Cancer Center). The G → A point mutation that results in the substitution of lysine for glutamic acid at amino acid 17 was introduced using Quick-Change Site-Directed Mutagenesis (Stratagene, La Jolla, CA, USA). The nucleotide change was confirmed by direct sequencing.

### Transient transfection and western blotting

The A375 human melanoma cell line was seeded the day before transfection on six-well tissue culture plates. Cells were transfected with indicated plasmids and Fugene HD Transfection Reagent (Roche, Indianapolis, IN, USA) as per the manufacturer's instructions. Eight hours after transfection the media was replaced. After 48 h transfection, media was replaced with media containing either 5 or 0% serum (‘Serum-free’). Twenty-four hours later the cells were harvested, washed with PBS, and resuspended in lysis buffer containing 1% Triton X-100, 50 mm HEPES, pH 7.4, 150 mM NaCl, 1.5 mM MgCl_2_, 1 mM EGTA, 100 mM NaF, 10 mM Na pyrophosphate, 1 mM Na_3_VO_4_, 10% glycerol, and supplemented with complete Protease Inhibitor Cocktail tablet (Roche). Protein isolation and western blotting were performed using the standard techniques (Hennessy *et al*, 2007). Antibodies against HA tag (Covance, Princeton, NJ, USA), Phospho-AKT Ser473 (C terminus), Phospho-AKT Thr308 (activation loop), and AKT3 (Cell Signaling Technology, Danvers, MA, USA) were used. The Phospho-AKT antibodies, which are designated above by the relevant residues in AKT1, also recognise the corresponding residues in AKT2 (Ser474, Thr309) and AKT3 (Ser472, Thr305).

## Results

### Detection of *AKT1 E17K* and *AKT3 E17K* mutations in melanoma

**Figure 1:**
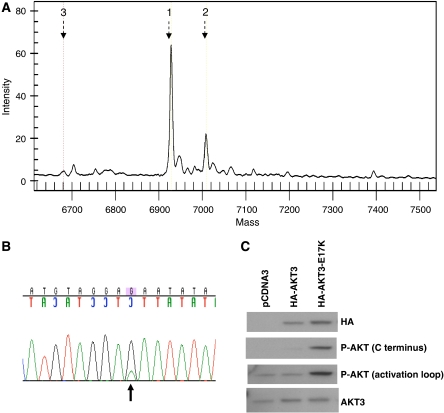
*AKT3 E17K* mutation in melanoma. (**A**) Mass spectroscopy-based detection of *AKT3 E17K* mutation in a human melanoma clinical specimen. Peaks correlating with wild-type *AKT3* (‘1’) and mutant *AKT3* (‘2’) are indicated. (‘3’=predicted mass of unincorporated primer). Only the wild-type peak was seen in normal tissue from the same patient (Supplementary Figure 1). (**B**) Confirmatory Sanger sequencing of tumour analysed by mass spectroscopy-based method in (**A**). The missense substitution resulting in the E17K mutation is indicated with an arrow. (**C**) Western blotting analysis of A375 human melanoma cells transfected with empty control vector (‘pcDNA3’), HA-tagged wild-type AKT3 (‘HA-AKT3’), and HA-tagged mutant AKT3 (‘HA-AKT3 E17K’). Results shown are for cells growing under normal tissue culture conditions.

We analysed melanoma clinical specimens for the presence of mutations in *AKT1*, *AKT2*, and *AKT3* that result in the *E17K* mutation identified previously in breast, ovarian, and colorectal cancers. We used mass spectroscopy-based mutation detection, which previously has been demonstrated to be a sensitive method for detecting specific point mutations in clinical specimens and cell lines (Thomas *et al*, 2007). Genomic DNA was analysed from 137 clinical specimens. Specimens were primarily from metastatic lesions, including soft tissue (*n*=16), lymph nodes (*n*=77), and parenchymal metastases (*n*=28); primary melanomas (*n*=16) were also analysed. We identified two lymph node metastases (from a single patient) that had the *AKT3 E17K* mutation (Figure 1A). This mutation was not present in the DNA from normal tissue of the same patient (Supplementary Figure 1). One lymph node metastasis from a different patient harboured the *AKT1 E17K* mutation. The *AKT3* and *AKT1E17K* mutations were confirmed by conventional Sanger sequencing (Figure 1B, and Supplementary Figure 2).

We performed a similar analysis on genomic DNA isolated from 65 human melanoma cell lines (Supplementary Figure 3). We did not identify any cell lines with *AKT1 E17K* or *AKT2 E17K* mutations. However, we identified two cell lines with *AKT3 E17K* mutations, WM46 and D40. Both cell lines were derived from metastatic lesions. It is unlikely that these cell lines were cross-contaminated or originated from the same patient, as they were derived in laboratories in different hemispheres, and the cell lines differed in the genetic sequences for other genes, including *MC1R* and *CDK4* (MAD and KS-H, unpublished data) (Landi *et al*, 2006).

### Activation of AKT3 by the E17K mutation

Previous studies demonstrated that expression of AKT1 E17K protein in NIH 3T3 cells increased AKT phosphorylation in those cells (Carpten *et al*, 2007). To determine whether the *AKT3 E17K* mutation results in a similar phenotype, we generated mammalian expression vectors with HA-tag-fused *AKT3* (wild type) and HA-tag-fused *AKT3 E17K*. These vectors, as well as empty control vector, were transiently transfected in the A375 human melanoma cell line. The A375 cell line was derived from a lymph node metastasis, the same anatomic setting as the specimens that harboured the *AKT3 E17K* mutation(Kozlowski *et al*, 1984). The A375 cells do not have an activating mutation of *AKT*, *PIK3CA*, or *N-ras*, and express PTEN protein (MAD and KS-H, unpublished data). Expression of AKT3 E17K in A375 cells increased AKT phosphorylation, as compared to transfection with the control vectors or wild-type AKT3, under normal tissue culture conditions (Figure 1, Panel C). Similar results were also seen when the cells were grown in the absence of growth factors (Supplementary Figure 4).

## Discussion

The PI3K signalling pathway is a critical regulator of many cellular processes that contribute to the aggressive nature of cancer, including proliferation, survival, invasion, and angiogenesis (Hennessy *et al*, 2005). We sought to determine if the recently identified activating mutation (*E17K*) in *AKT1*, which was initially detected in breast, colorectal, and ovarian cancers, and more recently in lung cancer, occurs in melanoma tumours and cell lines (Carpten *et al*, 2007; Kim *et al*, 2008; Malanga *et al*, 2008). In this study, we identified the same *AKT1 E17K* mutation reported previously in other cancers in the tumour from a melanoma patient. We also identified the analogous *E17K* mutation in *AKT3* in two different tumours (from the same patient) and in two human melanoma cell lines. Two previous studies failed to detect *AKT3* mutations in clinical specimens of breast (*n*=154), colorectal (*n*=155), ovarian (*n*=50), gastric (*n*=180), hepatocellular carcinoma (*n*=68), non-small cell lung cancer (*n*=157), and adult acute leukaemias (*n*=129) (Carpten *et al*, 2007; Kim *et al*, 2008). We have analysed an additional 547 primary breast cancers without detecting *AKT2* or *AKT3 E17K* mutations (Stemke-Hale *et al*, 2008). Thus, this study represents the first report of an *AKT3* mutation in human cancer.

Normal tissue from the patient with the *AKT3 E17K* mutation failed to show evidence of this mutation (Supplementary Figure 1). Thus, it is likely that the mutation was somatically acquired and was not present in the germline. Unfortunately, DNA was not available from the primary tumour of this patient, so it is unknown if the mutation was present only in metastases. Of note, in both of the tumour samples from this patient the observed prevalence of the *AKT3 E17K* mutation was ∼30% (Figure 1A), meaning that ∼60% of the analysed cells harbour the mutation if it is heterozygous. Visual review by an experienced dermatopathologist (AJFL) of H&E slides of these samples determined that the percentage of tumour cells in the specimens was close to 80%. It could be that only a portion of the tumour cells had the AKT3 mutation. Another explanation is that the lower percentage of tumour cells could be because of smaller infiltrating lymphocytes or other contaminating non-tumour cells that were in the deeper cuts used to extract DNA but not readily apparent in the H&E stain. Alternatively, the reason might be technical differences due to PCR amplification biases or because higher mass ions are discriminated against each other.

The discovery of an *AKT3* mutation in melanoma is consistent with previous data that suggest that this isoform of *AKT* may be particularly critical in this cancer. Stahl *et al* (2004) found that AKT3 protein, but not AKT1 or AKT2 protein, was increased in melanoma cell lines when compared to normal melanocytes. Transfection of siRNA against each AKT isoform in three different melanoma cell lines demonstrated that the AKT3 isoform was responsible for the majority of phosphorylated AKT in those cells (Stahl *et al*, 2004). While previous studies of small numbers of melanomas (*n*=20 and *n*=16, respectively) failed to identify mutations in *AKT* (Waldmann *et al*, 2001, 2002), copy-number increases of the region of chromosome 1 where *AKT3* is located have been reported (Thompson *et al*, 1995; Bastian *et al*, 2000; Curtin *et al*, 2005).

It will be important to determine the functional significance of the *AKT3 E17K* mutation. Preclinical models have demonstrated that the isoforms of *AKT* may have markedly different effects on cellular behaviours, including invasiveness and metastatic potential (Hutchinson *et al*, 2004; Irie *et al*, 2005). Others have demonstrated that although AKT1, AKT2, and AKT3 have several shared substrates, there are also proteins that are selectively regulated by individual isoforms (Brognard *et al*, 2007). A critical finding in the data presented here is the identification of two human cancer cell lines with the *AKT3 E17K* mutation. To date, we have tested a total of 178 human cancer cell lines for *AKT E17K* mutations, including the entire NCI60 cell line collection, as well as additional breast (*n*=41) and ovarian (*n*=27) cancer cell lines (KS-H, not presented). WM46 and D40 were the only cell lines found to harbour the *E17K* mutation in any *AKT* isoforms, and to our knowledge are the first human cell lines identified with any *AKT* mutation. These cell lines can be utilised to examine the function of the AKT3 E17K mutant protein in a cellular setting in which physiologically relevant compensatory changes have occurred.
