# Supplementary figures and images for: A novel AKT3 mutation in melanoma tumours and cell lines
Source: Br J Cancer. 2008 Sep 23;99(8):1265–8. doi: 10.1038/sj.bjc.6604637 (PMC2570525; doi:10.1038/sj.bjc.6604637)

## Slide 1
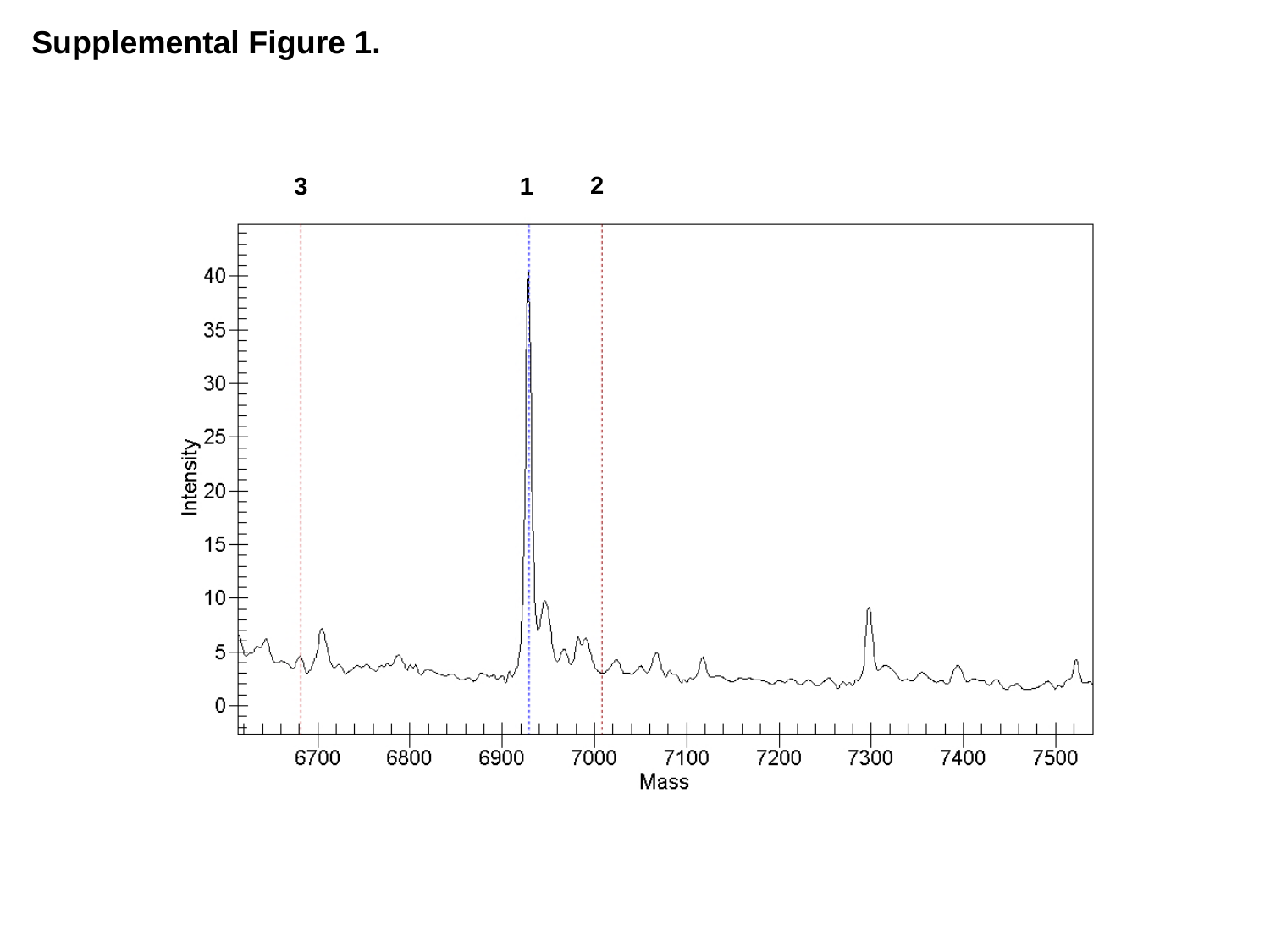

Supplemental Figure 1.
2
3
1

Supplement: Supplementary Figure 1 [file 6604637x1.ppt]

## Slide 1
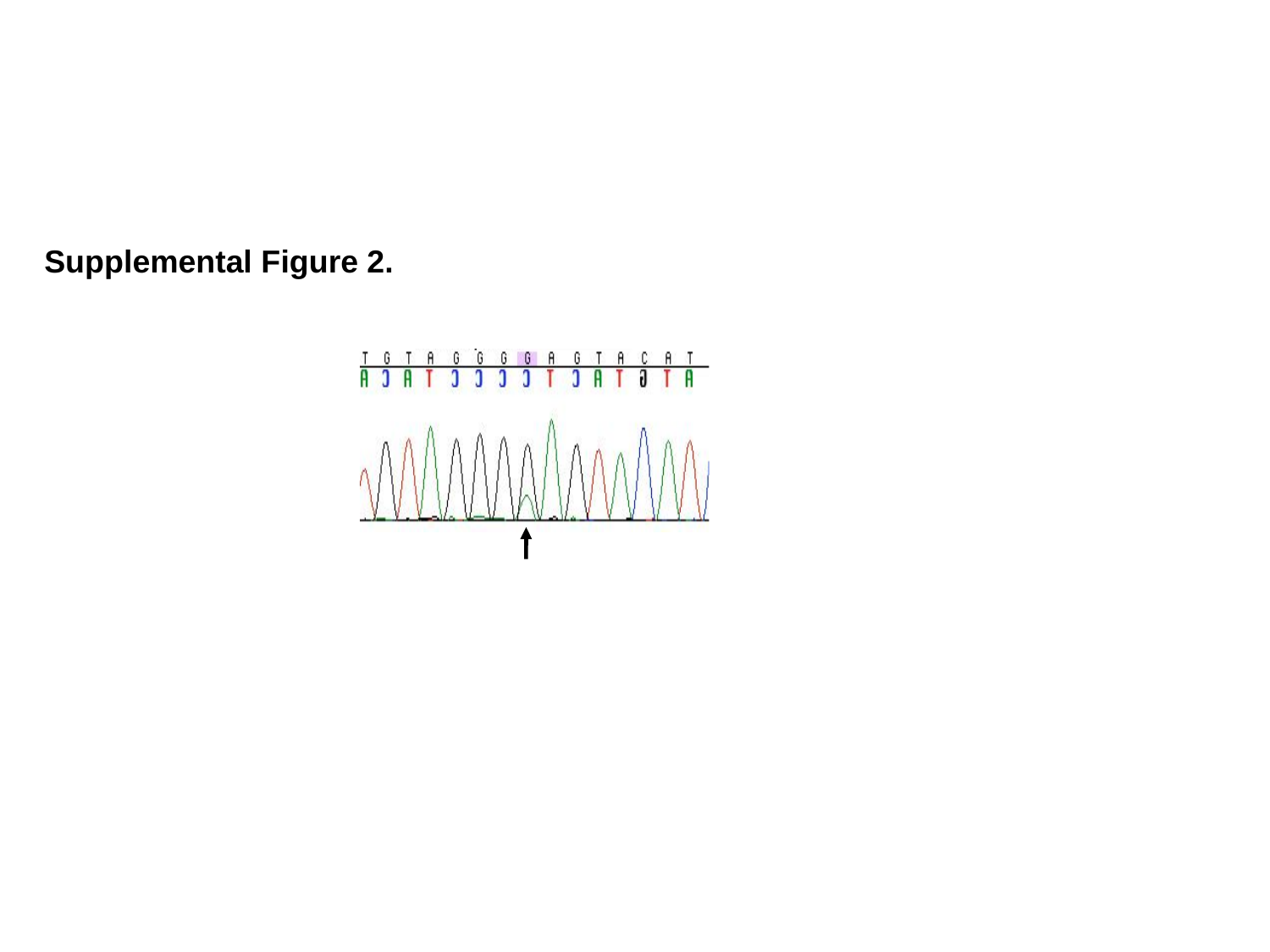

Supplemental Figure 2.

Supplement: Supplementary Figure 2 [file 6604637x2.ppt]

## Slide 1
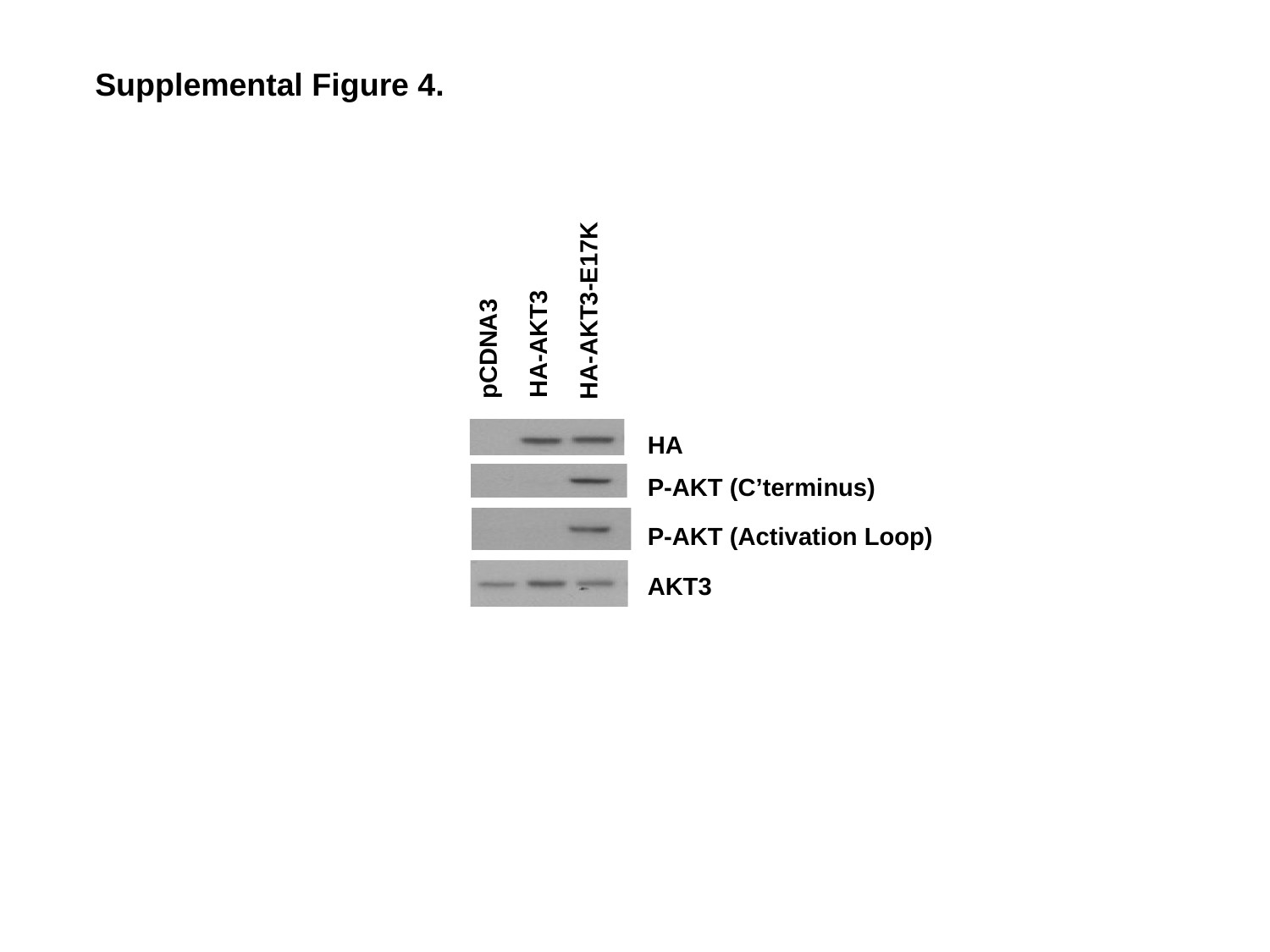

Supplemental Figure 4.
HA-AKT3-E17K
HA-AKT3
pCDNA3
HA
P-AKT (C’terminus)
P-AKT (Activation Loop)
AKT3

Supplement: Supplementary Figure 4 [file 6604637x4.ppt]
