# Supplementary material for: A novel AKT3 mutation in melanoma tumours and cell lines
Source: Br J Cancer. 2008 Sep 23;99(8):1265–8. doi: 10.1038/sj.bjc.6604637 (PMC2570525; doi:10.1038/sj.bjc.6604637)
Supplement: Supplementary Figure 3 [file 6604637x3.doc]

1205 Lu MEL 938 WM1552

451Lu MEWO WM1617

A11 MM466 WM164

A15 MM595 WM1727A

A2 MM649 WM1799

A2058 PA14 WM1819

A375 RPMI7951 WM239

A375SM SB2 WM239A

C8161 SKMEL 2 WM266-4

D24 SKMEL 28 WM278

D25 SKMEL 5 WM3211

D40 SKMEL 23 WM3268

DM4 SKMEL 37 WM3451

HS294T TXM-1 WM35

LOX IMVI UACC 257 WM46

M14 UACC 62 WM51

M19 UCSD 354L WM793

MALME 3M UCSD 242L WM852

MEL 526 WM115 WM88

MEL 624 WM1341B WM983A

MEL 888 WM1346 WM983B

WM1361A WM983C

**Supplemental Figure 3**
