# Supplementary material for: A novel AKT3 mutation in melanoma tumours and cell lines
Source: Br J Cancer. 2008 Sep 23;99(8):1265–8. doi: 10.1038/sj.bjc.6604637 (PMC2570525; doi:10.1038/sj.bjc.6604637)
Supplement: Supplementary Figures Legends [file 6604637x5.doc]

Supplemental Information for:

**A Novel *AKT3* Mutation in Melanoma Tumors and Cell Lines.**

Michael A. Davies, Department of Melanoma Medical Oncology, The University of Texas M. D. Anderson Cancer Center, 1515 Holcombe Blvd, Houston, Texas, 77030, mdavies@mdanderson.org (*corresponding author*) et al

**Figure Legends**

Supplemental Figure 1. Analysis of DNA of normal tissue from melanoma patient with *AKT3 E17K* mutation. Mass spectroscopy-based detection of *AKT3 E17K* mutation in a clinical specimen. Location of peaks correlating with wild-type *AKT3* (“1”) and mutant *AKT3* (“2”) are indicated. [“3” = predicted mass of unincorporated primer]. No significant peak at the mass corresponding to the mutant *AKT3* allele was detected, indicating that no mutation was present in this sample. Thus, it is likely that the *AKT3 E17K* mutation in this patient is patient was somatically acquired, and it is not a germline mutation.

Supplemental Figure 2. *AKT1 E17K* mutation in melanoma. Confirmatory Sanger sequencing results of a human melanoma found to have an *AKT1 E17K* mutation. The missense substitution resulting in the E17K mutation is indicated with an arrow.

Supplemental Figure 3. Human melanoma cell lines screened for *AKT1*, *AKT2*, and *AKT3 E17K* mutations.

Supplemental Figure 4. Expression of AKT3 E17K activates AKT in the absence of growth factors. Western blotting analysis of A375 human melanoma cells transiently transfected with empty control vector (“pcDNA3”), HA-tagged wild-type AKT3 (“HA-AKT3”), or HA-tagged mutant AKT3 (“HA-AKT3 E17K”). 48 hours after transfection the cells were switched to serum-free media, then were lysed after 24 hours. Expression of the AKT3 E17K maintained AKT phosphorylation in the absence of growth factors. Western blotting results for HA, Phospho-AKT (C’-Terminus), Phospho-AKT (Activation Loop), and Total AKT3 are shown.
